# Larch Bark as a Formaldehyde Scavenger in Thermal Insulation Panels

**DOI:** 10.3390/polym12112632

**Published:** 2020-11-10

**Authors:** Marius Cătălin Barbu, Yasmin Lohninger, Simon Hofmann, Günther Kain, Alexander Petutschnigg, Eugenia Mariana Tudor

**Affiliations:** 1 Forest Products Technology and Timber Construction Department, Salzburg University of Applied Sciences, Markt 136a, 5431 Kuchl, Austria; cmbarbu@unitbv.ro (M.C.B.); yasmin.lohninger@gmx.at (Y.L.); s.hofmann@e-w-enture.de (S.H.); gkain.lba@fh-salzburg.ac.at (G.K.); alexander.petutschnigg@fh-salzburg.ac.at (A.P.); 2 Faculty of Furniture Design and Wood Engineering, Transilvania University of Brasov, B-dul. Eroilor nr. 29, 500036 Brasov, Romania; 3 Higher Technical College Hallstatt, Lahnstraße 69, 4830 Hallstatt, Austria

**Keywords:** larch bark, free formaldehyde, formaldehyde emissions, insulation panels

## Abstract

The aim of this study is to investigate the formaldehyde content and emissions of bark-based insulation panels bonded with three types of adhesives: urea formaldehyde, melamine urea-formaldehyde, and tannin-based adhesives. These panels were produced at two levels of density—300 and 500 kg/m^3^—and a thickness of 20 mm, and the influence of the adhesive amount and type on the formaldehyde emissions and content was measured. Other mechanical and physical properties such as modulus of rupture, modulus of elasticity, internal bond, and dimensional stability were also scrutinized. With one exception, all the panels belonged to the super E0 classification for free formaldehyde content (perforator value ≤1.5 mg/100 g oven dry mass of panels). The measurements using the desiccator method for formaldehyde emissions assigned all the testing specimens in the F **** category for low-emission panels according to the Japanese International Standards.

## 1. Introduction

A feasible building culture includes environmentally friendly alternatives for heat insulation materials. Natural insulation, produced from renewable resources, gained increased popularity in the last few decades. The challenge of developing eco-friendly building materials leads to products that are designed for well-being and health [1]. During this time, insulating materials developed to such a high range that they met most all of customers’ expectations. However, only one insulating material cannot fulfill all requirements, and the ecological aspects are not always reflected in the most expensive products [2]. Some properties such as low diffusion resistance, moisture resistance, good impact sound insulation, high compressive strength, exclude each other [3]. Regarding the market for heat insulation materials, the main products are still polystyrene or mineral wool, which are nowadays competing with high-performance new and recyclable products [4,5]. The manufacturing of nature-based heat insulation materials involves less fossil fuel energy [6] and less energy-intensive processes [7]. The recycling of building waste (thermal insulation materials, synthetic or mineral) is problematic. Their degradation is very slow and might generate toxic substances [5]. Insulating materials are manufactured for different applications and with particular properties, according to the final use [3]. Of these properties, the most important one is thermal conductivity, complemented by other properties such as compressive strength and fire behavior, all of them in a direct relationship with the end use [8]. The hazards correlated with the manufacturing of thermal insulation panels have increased due to chemical substances being included in the fabrication process [9]. Moreover, the contaminant emission standards for building materials have been significantly extended [1]. Therefore, the impact of exposure to unhealthy materials on humans during and after the preparation process must be assessed. The tendency of modern work and lifestyle shows that people spend about 90% of their daily life in indoor areas [10]. Air pollution, especially indoors, is responsible for the high rate of lung cancer [1]. Formaldehyde is a well-known air pollutant and the first carcinogen in humans [11]. Reducing formaldehyde levels in indoor and outdoor applications is an important topic nowadays [12]. One natural by-product of the wood industry—namely, tree bark—can be successfully used for the reduction in formaldehyde emissions in wood-based composites—e.g., spruce bark (*Picea abies*) [13]. Jahanshaei et al. [14] developed an eco-friendly tannin-phenol formaldehyde resin to decrease the formaldehyde level of wood composites [15] using a bark alkaline extractive from lodgepole pine (*Pinus contorta*) to produce bio-based melamine formaldehyde resins. Benuang bark (*Octomeles sumatrana*/BN) and duabanga bark (*Duabanga moluccana*/DB) can act as filler in phenol-formaldehyde [16] adhesives. Red cedar bark (*Thuja plicata*) was analyzed in compounds with isocyanates (pMDI) resins [17,18]; the authors analyzed the influence of beech bark (*Fagus sylvatica*) in adhesive mixtures used in plywood (Ply) and [19] carried out research on the effects of using birch bark (*Betula pendula*) particles with various dimensions as a filler for urea-formaldehyde resin in Ply, similar to the study of [20], who analyzed the role of walnut, chestnut, fir, and spruce bark flours instead of wheat flour in Ply production. The radical scavenging activity of the outer and inner bark of alder (*Alnus glutinosa* L.), oak (*Quercus robur* L.), and pine (*Pinus sylvestris* L.) was studied by [12,21]; the authors studied the capacity of larch bark (*Larix decidua* Mill.) for formaldehyde removal in wood adhesives. Larch bark could be used as thermal insulating material for buildings, with an average 20% lower thermal conductivity than wood [22]. Larch inner bark is on average 35% lighter than larch wood (300 kg/m^3^) and is therefore well suited to be used for thermal insulation purposes [23]. The aim of this study is to analyze the role of larch bark as a formaldehyde scavenger in thermal insulation boards when bonded with a tannin-based adhesive, urea-formaldehyde (UF), and melamine urea-formaldehyde (MUF).

## 2. Materials and Methods

The larch bark was sourced from Graggaber Sawmill (Unternberg, Austria). The bark was dried in a vacuum kiln dryer (Brunner–Hildebrand High VAC-S, HV-S1, Hannover, Germany) from a 50% to 6% moisture content. The drying temperature was 60 °C at a pressure of 200–250 mbar. The bark was subsequently crushed in a 4-spindle shredder (RS40) at the Untha Co. (Kuchl, Austria), and repeatedly screened using a sieve shaker Retsch AS 200 (Haan, Deutschland) to obtain 6–10 mm particles.

The bark-based insulation panels (50 × 50 cm), with two levels of density—300 and 500 kg/m^3^—and a thickness of 20 mm, were prepared with 8%, 12%, and 16% adhesives as follows: UF (Dynea Prefere 10F102, Krems, Austria) with 1% ammonium sulphate hardener; MUF type Prefere 4561 (Dynea, Krems, Austria) with 1% hardener type Prefere 5011 (Dynea, Krems, Austria) and a tannin-based adhesive (Table 1). The latter was prepared with an extract powder from Colatan GT5 Quebracho tannin (*Schinopsis Lorentzii and Schinopsis balansae*) from Christian Markmann Co. (Hamburg, Germany), reinforced with 5% resorcinol, hexa-methylenetetramine (hexamine) from Merck Schuchardt, Hohenbrunn, Germany (C 99%) and 32% sodium hydroxide solution from Carl Roth (Karlsruhe, Germany). Amounts of 50% tannin extract powder and 50% water were stirred with 700 and 1500 rpm in a mechanical mixer. Then, 10% of hexamine was added to adjust the pH value of the mixed solution to 9 using a sodium hydroxide solution. The blending of bark particles with the adhesives was conducted in a ploughshare mixer ENT type WHB-75 for 10 min. The panels were pressed at 180 °C for 8 min using a laboratory press, Höfer HLOP 280 (Taiskirchen, Austria), with the following adjustment tensile of 0.97 at a measurement range of 4000–20,000 N. The panels were pressed with a press factor of 24 s/mm (significantly higher compared to industry). The maximal pressure was 30 bar and the minimal was 10 bar. The press cycle included a pre-heating stage at 19 mm for 30 s, then the boards were pressed for 7.5 min.

The formaldehyde testing methods can be divided into three categories, according to [24]: total amount testing method—e.g., perforation; static emission testing method—e.g., desiccator; and dynamic emission testing method—e.g., chamber. For this study, the perforator method, mostly used in industry, and the desiccator method [25] were chosen. This study follows the previous research of [12,22,26,27].

The testing of formaldehyde content was carried out according to [28]. Small specimens (25 × 25 mm, 110 g each batch) were extracted by means of boiling toluene and then transferred into distilled or demineralized water. The formaldehyde emission was sampled through perforation in water and analyzed photometrically with the acetylacetone method. The perforator value depends on the moisture content of the specimens [29], which was determined according to [30]. The perforator values were corrected to boards conditioned to a moisture content of 6.5%. This method demands a running time of 3 h and is used mostly for production control in the wood-based panels industry [29].

One of the testing methods used to determine the formaldehyde emission from insulating boards was made according to [31]. Prior to this test, the 150 ± 1 × 50 ± 1 mm specimens were conditioned for one week to a constant mass in a climate chamber at 20 ± 2 °C and a relative air humidity of 65 ± 5%. Eight samples from each board were cut carefully and the edges were sealed with paraffin. Then, the test pieces were placed for 24 h at 20 °C in a desiccator filled with 300 mL of deionized water underneath the samples. The quantity of formaldehyde released was determined from the concentration of formaldehyde absorbed in deionized water based on the Hantzsch reaction between formaldehyde and ammonium ions and acetylacetone with an output of diacetyldihydrolutidine. The concentration of formaldehyde in the solution was determined photometrically [31]. The mechanical properties were determined by employing a universal testing machine, Zwick Roell 250 (Ulm, Germany), for the measurement of the modulus of rupture and the modulus of elasticity according to [32], the internal bond according to [33], and the thickness swelling and water absorption according to [34].

The values of thermal conductivity were not reported in this study, because they were described in the research of [27].

## 3. Results and Discussion

### 3.1. Free Formaldehyde Content and Formaldehyde Emission

**Figure 1:**
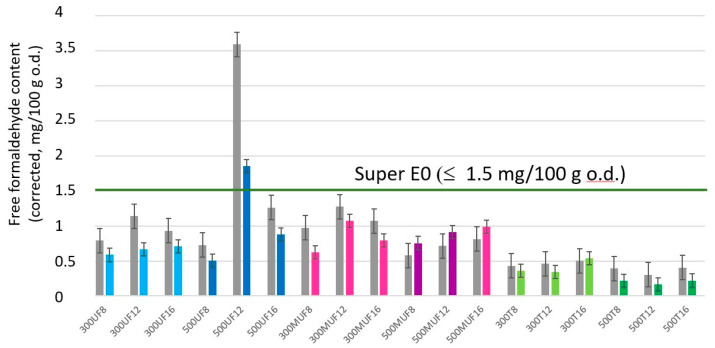
Free formaldehyde content (mg/100 g o.d.) for both the measured and (moisture-)corrected values for larch-bark insulating boards (300 and 500 kg/m^3^)**.**

With only one exception (sample bonded with 12% UF and 500 kg/m^3^), the mean values of free formaldehyde content corrected to 6.5% moisture content of the larch bark insulating boards glued with UF and tannin, determined by a perforator test, are included in the super E0 category, being lower than 1.5 mg/100 g oven dry (o.d.) (Figure 1). There is no significant difference between the free formaldehyde content of specimens bonded with UF or MUF for both density ranges of 300 and 500 kg/m^3^. In the case of tannin-based adhesive, the values did not reach more than 0.5 mg/100 g oven dry mass of panels.

At a density of 300 kg/m^3^, the highest amount of free formaldehyde was detected for the sample bonded with 12% UF (1.07 mg/100 g o.d.) and the lowest value for the sample bonded with 12% tannin-based adhesive (0.34 mg/100 g o.d.). This trend is due to the fact that formaldehyde reacts with tannins and produces polymerization through methylene bridges to reactive positions of the flavonoid molecules [35].

It is interesting how an amount of 12% UF and MUF adhesive results in higher levels of free formaldehyde content compared to a percentage of 16%. In the latter case, the measured values are all between 0.5 and 1.5 mg/100 g o.d. Urea acts indeed as a formaldehyde scavenger mingled with the polyphenols in the larch bark, the tannins. The reason for larch bark being able to retain formaldehyde and diminishing its emission [36] is due to its condensed tannin content of 56 ± 6 mg/kg of dry European larch bark (*Larix decidua* Mill.) [37]. The values of free formaldehyde content lower than 0.5 mg/100 g for the tannin-bonded panels are justified by the reaction of the hardener hexamine, which does not decompose to formaldehyde and ammonia due to the mixing with chemical species with very reactive nucleophilic sites, such as condensed flavonoid tannins. The results of this reaction are the building of aminomethylene bridges before any chance to yield formaldehyde [38]

**Figure 2:**
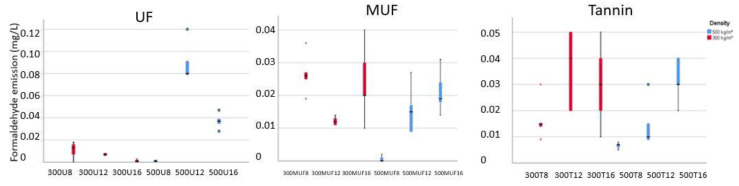
Formaldehyde emission (mg/L) of the larch bark-based insulation boards 300 and 500 kg/m^3^ bonded with UF, MUF, and tannin-based adhesives.

The formaldehyde emission measured for the samples of larch bark-based insulation boards are all below 0.1 mg/L (Figure 2). That means that all the boards can be included in the F **** classification. From the four levels of formaldehyde limits defined by the Japanese International Standards (JIS), F **** is the most rigorous one, with an average value of less than 0.3 mg/L. It is also known as zero formaldehyde emission (≤0.3 mg/L) and is similar to the formaldehyde emission from natural wood (0.1–0.3 mg/L), determined through the desiccator method [25].

The same tendency observed for the free formaldehyde at 12% UF at 500 kg/m^3^ (Figure 2) was detected also for the formaldehyde emission of the same sample (three times bigger compared with the sample with 16% UF). For the other samples, the difference in glue percentage from 12% to 16% is interesting to observe because the increased adhesive amount does not significantly influence the level of formaldehyde emission (from 0 to 0.03 mg/L).

### 3.2. Mechanical and Physical Properties of Larch Bark Insulating Panels

Aside from the capacity of tannins to act as a formaldehyde scavenger, their effect on the mechanical and physical properties was determined in this study. The values for the modulus of rupture (MOR); modulus of elasticity (MOE); internal bond (IB); the dimensional stability—thickness swelling (TS) and water absorption (WA) after 24 h immersion in water at 20 °C—are presented in Table 2.

### 3.3. Moduli of Rupture and Elasticity

**Figure 3:**
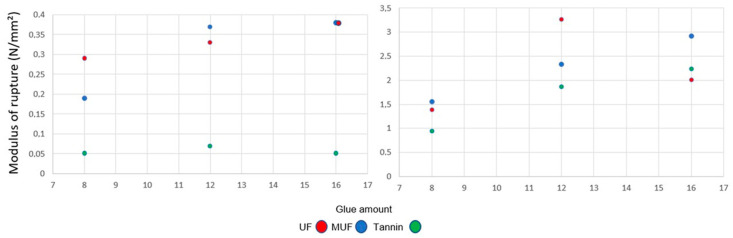
Modulus of rupture at three levels of glue amount for 300 kg/m^3^ (**left**) and 500 kg/m^3^ (**right**).

The dependence of MOR on density is clearly reflected in Figure 3. For the panels with 300 kg/m^3^, the MOR did not reach more than 0.4 N/mm^2^. The lowest values of MOR were measured for the samples bonded with tannin-based adhesive for all percentages of glue amounts. There is no significant influence on MOR considering the use of UF or MUF.

MOR increases at 500 kg/m^3^, being almost nine times higher than that of the panels with a density of 300 kg/m^3^. In this case, at 8% glue the values of MOR in the case of bonding with UF or MUF are quite similar. For 12% UF, the MOR of the panels is 3.27 N/mm^2^ and 30%, 40% higher compared with the 12% MUF and 12% tannin-based adhesive. An increase in the adhesive amount above 12% does not improve the MOR, with all the measured values being under 3 N/mm^2^.

**Figure 4:**
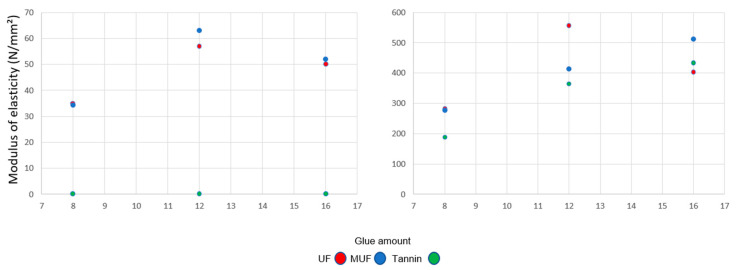
Modulus of elasticity at three levels of glue amount for 300 kg/m^3^ (**left**) and 500 kg/m^3^ (**right**).

The trend described for MOR is similar in the case of the measured values for MOE at both densities, with the exception of the tannin-based samples with a density of 300 kg/m^3^, which are 0 N/mm^2^ for all glue percentages (Figure 4). Twelve percent UF and MUF does not definitely influence the MOE. At 16% adhesive, the MOE is almost equal for UF and MUF. With increased density (500 kg/m^3^), the values of MOE increase too, from a minimum of 200 N/mm^2^ (8% tannin-based adhesive) to 560 N/mm^2^ (12% UF).

### 3.4. Internal Bond

**Figure 5:**
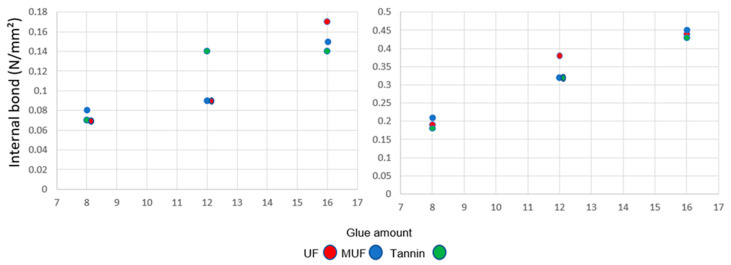
Internal bond at three levels of glue amount for 300 kg/m^3^ (**left**) and 500 kg/m^3^ (**right**).

The values of IB (Figure 5) are comparable for UF, MUF, and tannin for a glue amount of 8% and a density of 300 kg/m^3^ (up to 0.08 N/mm^2^). At 12% glue, UF and MUF perform analogously (0.09 N/mm^2^), and for the tannin-bonded panels an IB of 0.14 N/mm^2^ was recorded (the same value at 16% glue). There is a slight increase for 16% UF (0.17 N/mm^2^) compared to 0.15 and 0.14 N/mm^2^ for MUF and tannin adhesive, respectively. For the 500 kg/m³ density, at two levels of glue amount, 8% and 16%, the influence of adhesive is not significant. Only at 12% resin is there a significant difference between the values for MUF, tannin (0.32 N/mm^2^), and UF (0.38 N/mm^2^), which is at the same level (0.35 N/mm^2^) as the P2 particleboard (used for furniture components, partitions, flooring base, packaging, and sandwich panels) according to [39].

### 3.5. Thickness Swelling and Water Absorption after 24 h

**Figure 6:**
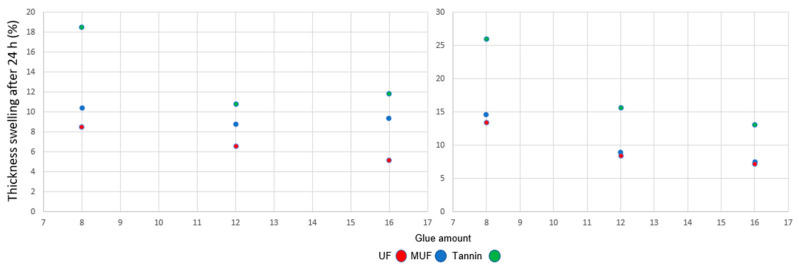
Thickness swelling after 24 h at three levels of glue amount for 300 kg/m^3^ (**left**) and 500 kg/m^3^ (**right**).

The resin type significantly influences the TS and WA [27,40] reported about the increase in TS and WA when using tannin-based adhesives. For both density ranges, all the tannin-bonded samples recorded the highest values for thickness swelling (Figure 6) after 24 h of water immersion (18.5%, 11%, and 12%) for 8%, 12%, and 16% glue amounts (300 kg/m^3^) and 26%, 16%, and 13% at a density of 500 kg/m^3^. The testing specimens bonded with UF had the lowest thickness swelling—under 10% for a density of 300 kg/m^3^ and under 14.6% for 500 kg/m^3^.

**Figure 7:**
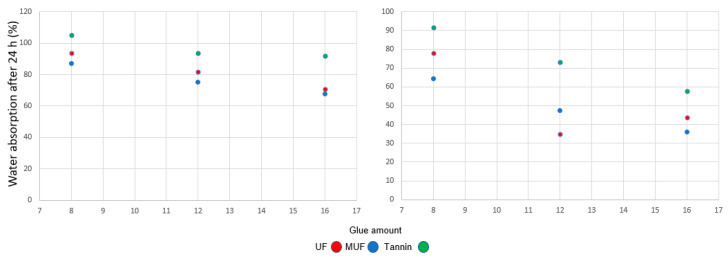
Water absorption after 24 h at three levels of glue amount for 300 kg/m^3^ (**left**) and 500 kg/m^3^ (**right**).

More than 80% WA was measured for the tannin-glued samples for the three different glue amounts (Figure 7). With UF and MUF, the WA decreases under 100% at a density of 300 kg/m^3^, with a minimum of 68% for the MUF-bonded samples. When the density increases with 200 kg/m^3^, the water absorption of the tannin-bonded specimens is from 60% (16% glue) to 90% (8% glue), from 35% (12% glue) to 87% (8% glue) in the case of UF, and from 36% (12% glue) to 64% (8% glue). A lower adhesive amount has a direct influence on the dimensional stability of the insulation panels. The TS and WA improve at glue percentages of 12% and 16%, respectively.

## 4. Conclusions

The high amounts of tannins in the larch bark (about 30%) [37,41] determine a decrease in formaldehyde emissions due to the ability of larch bark to retain formaldehyde and diminish its emission due to its condensed tannin content [36]. Jahanshaei et al. [14] stated that the condensed polyflavonoid tannins (polyphenols) of the bark can withstand reactions with formaldehyde in both acid and alkaline media. With the exception of the panels bonded with 12% UF at a density of 500 kg/m^3^ with a measured perforator value (free formaldehyde content) of 1.85 mg/100 g o.d., which belongs to the E0 category (≤3.5 mg/100 g o.d.), the rest of the insulation boards are included in the super E0 category (≤1.5 mg/100 g o.d.).

Regarding the formaldehyde emission measured with desiccator method, all the investigated values were lower than 0.1 mg/L, which is three times lower than the limit for the F **** JIS classification for formaldehyde emissions. It results that the bark-based insulation panels present an increased eco-efficiency compared to wood, which has a level of 0.1–0.3 mg/L formaldehyde emission.

MOR is not one of the strengths of these panels. It was a maximum of 3 N/mm^2^ measured for the panel bonded with UF, while other values did not reach more than 2 N/mm^2^ when the other adhesives, MUF and tannin, were involved. Nevertheless, light bark particleboard will be probably used for non-structural applications where the MOR obtained is sufficient.

The MOE was zero for the tannin samples at a 300 kg/m^3^ density and reached 560 N/mm^2^ for the panels with 12% UF and 500 kg/m^3^. Both MOR and MOE are at a down level for all typed of bark-based insulation panels. The maximal value measured for the IB was 0.44 N/mm^2^, which is in accordance with the results of [27].

The dimensional stability was improved by the use of UF and MUF adhesives.

The use of bark for insulation panels ensured decreased levels of free formaldehyde content and formaldehyde emissions at a level which is not reachable when using only wood particles glued with the same adhesive types and amounts. The recyclability of these insulation panels compared with similar products, principally mineral-based, recommend this alternative and sustainable raw material tree bark for eco-friendly applications, especially for indoor purposes.

The specific impact on the reduction in free formaldehyde and formaldehyde content of the larch bark recommend this raw material for further research in green construction solutions.

## Figures and Tables

**Table 1:** Experimental design with the factors density and resin content (based on the oven-dried weight of bark particles).

Board	Density	Glue	Glue	Moisture
	(kg/m^3^)	Type	Amount (%)	Content (%)
UF1	500	UF	8	8.7
UF2	500	UF	12	10.1
UF3	500	UF	16	8.7
UF4	300	UF	8	8.4
UF5	300	UF	12	9.6
UF6	300	UF	16	8.2
MUF1	500	MUF	8	4.2
MUF2	500	MUF	12	4.4
MUF3	500	MUF	16	4.8
MUF4	300	MUF	8	9.2
MUF5	300	MUF	12	7.7
MUF6	500	MUF	8	8.4
T1	500	Tannin	12	5.8
T2	500	Tannin	16	7.3
T3	300	Tannin	8	9.9
T4	300	Tannin	12	7.6
T5	300	Tannin	16	8.4a
T6	300	Tannin	16	5.9

**Table 2:** Mechanical and physical properties of the larch-bark insulation panels (standard deviation in parentheses).

Sample	MOR (*N*/mm^2^)	MOE (*N*/mm^2^)	IB (*N*/mm^2^)	TS 24 h (%)	WA 24 h (%)
300UF8	0.29 (0.03)	35.02 (3.62)	0.07 (0.01)	8.5 (0.81)	93.71 (2.02)
300UF12	0.33 0.04)	57.15 (12.98)	0.09 (0.02)	6.54 (1.9)	81.75 (1.2)
300UF16	0.38 (0.02)	50.22 (5.35)	0.17 (0.04)	5.21 (1.98)	70.86 (0.56)
500UF8	1.39 (0.17)	283.18 (29.95)	0.19 (0.02)	14.64 (0.93)	78.08 (1.68)
500UF12	3.27 (0.53)	556.78 (26.36)	0.38 (0.04)	8.42 (1.35)	34.79 (3.31)
500UF16	2.01 (0.09)	403.05 (73.24)	0.44 (0.03)	7.18 (1.1)	43.8 (7.29)
300MUF8	0.19 (0.07)	34.36 (2.15)	0.08 (0.02)	10.41 (1.71)	87.09 (4.35)
300MUF12	0.37 (0.02)	63.16 (6.83)	0.09 (0)	8.72 (1.9)	75.23 (3.43)
300MUF16	0.38 (0.06)	52 (5.92)	0.15 (0.02)	9.38 (3.04)	67.55 (3.92)
500MUF8	1.55 (0.01)	278.16 (7.03)	0.21 (0)	13.36 (2.81)	64.21 (3.96)
500MUF12	2.33 (0.09)	414.41 (13.68)	0.32 (0.03)	8.86 (1.24)	47.48 (5.99)
500MUF16	2.92 (0.61)	511.28 (53.99)	0.45 (0.06)	7.51 (0.61)	36.14 (4.89)
300T8	0.05 (0.01)	0 (0)	0.07 (0.01)	18.47 (2.34)	105.3 (1.08)
300T12	0.07 (0)	0 (0)	0.14 (0.01)	10.79 (1.59)	93.71 (2.23)
300T16	0.05 (0)	0 (0)	0.14 (0.01)	11.85 (1.75)	91.81 (0.91)
500T8	0.94 (0.07)	187.72 (16.32)	0.18 (0.01)	25.89 (1.74)	91.53 (2.16)
500T12	1.87 (0.09)	363.59 (11.05)	0.32 (0.03)	15.59 (1.42)	73.41 (6.16)
500T16	2.24 (0.39)	432.26 (41.71)	0.43 (0.01)	13.06 (1.34)	57.86 (8.84)
